# Left and right ventricular dysfunction in patients with COVID-19-associated myocardial injury

**DOI:** 10.1007/s15010-020-01572-8

**Published:** 2021-01-30

**Authors:** Stéphanie Bieber, Angelina Kraechan, Johannes C. Hellmuth, Maximilian Muenchhoff, Clemens Scherer, Ines Schroeder, Michael Irlbeck, Stefan Kaeaeb, Steffen Massberg, Joerg Hausleiter, Ulrich Grabmaier, Mathias Orban, Ludwig T. Weckbach

**Affiliations:** 1 Medizinische Klinik und Poliklinik I, Klinikum der Universitaet Muenchen, Ludwig-Maximilians-University, Marchioninistraße 15, 81377 Munich, Germany; 2 COVID-19 Registry of the LMU Munich (CORKUM), University Hospital, LMU Munich, Munich, Germany; 3 Medizinische Klinik und Poliklinik III, Klinikum der Universitaet Muenchen, Ludwig-Maximilians-University, Munich, Germany; 4 Max Von Pettenkofer Institute and Gene Center, Virology, National Reference Center for Retroviruses, Faculty of Medicine, LMU Muenchen, Munich, Germany; 5 DZIF (German Center for Infection Research), Partner Site, Munich, Germany; 6 Department of Anaesthesiology, Ludwig-Maximilians-University, Munich, Germany; 7 DZHK (German Centre for Cardiovascular Research), Partner Site, Munich, Germany; 8 Institute of Cardiovascular Physiology and Pathophysiology, Biomedical Center, Ludwig-Maximilians-University, Planegg-Martinsried, Germany

**Keywords:** COVID-19, Myocardial injury, Heart failure, Three-dimensional echocardiography, Global longitudinal strain

## Abstract

**Purpose:**

SARS-COV-2 infection can develop into a multi-organ disease. Although pathophysiological mechanisms of COVID-19-associated myocardial injury have been studied throughout the pandemic course in 2019, its morphological characterisation is still unclear. With this study, we aimed to characterise echocardiographic patterns of ventricular function in patients with COVID-19-associated myocardial injury.

**Methods:**

We prospectively assessed 32 patients hospitalised with COVID-19 and presence or absence of elevated high sensitive troponin T (hsTNT+ vs. hsTNT-) by comprehensive three-dimensional (3D) and strain echocardiography.

**Results:**

A minority (34.3%) of patients had normal ventricular function, whereas 65.7% had left and/or right ventricular dysfunction defined by impaired left and/or right ventricular ejection fraction and strain measurements. Concomitant biventricular dysfunction was common in hsTNT+ patients. We observed impaired left ventricular (LV) global longitudinal strain (GLS) in patients with myocardial injury (-13.9% vs. -17.7% for hsTNT+ vs. hsTNT-, *p* = 0.005) but preserved LV ejection fraction (52% vs. 59%, *p* = 0.074). Further, in these patients, right ventricular (RV) systolic function was impaired with lower RV ejection fraction (40% vs. 49%, *p* = 0.001) and reduced RV free wall strain (-18.5% vs. -28.3%, *p* = 0.003). Myocardial dysfunction partially recovered in hsTNT + patients after 52 days of follow-up. In particular, LV-GLS and RV-FWS significantly improved from baseline to follow-up (LV-GLS: -13.9% to -16.5%, *p* = 0.013; RV-FWS: -18.5% to -22.3%, *p* = 0.037).

**Conclusion:**

In patients with COVID-19-associated myocardial injury, comprehensive 3D and strain echocardiography revealed LV dysfunction by GLS and RV dysfunction, which partially resolved at 2-month follow-up.

**Trial registration:**

COVID-19 Registry of the LMU University Hospital Munich (CORKUM), WHO trial ID DRKS00021225.

**Supplementary Information:**

The online version contains supplementary material available at 10.1007/s15010-020-01572-8.

## Introduction

Since the outbreak of the SARS-CoV-2 global pandemic in December 2019, COVID-19 has been shown to represent a multi-organ disease with suspected impact on myocardial function. First reports of hospitalised COVID-19 patients from Wuhan, China, have found myocardial injury with elevated high-sensitive troponin (hsTNT) levels. COVID-19-related myocardial injury was associated with higher admission rate on intensive care unit (ICU) and mortality [1–3]. Although the prevalence of myocardial injury has been estimated as high as 30% in hospitalised COVID-19 patients, its aetiology is heterogeneous and not fully understood [4].

Previous echocardiographic reports of COVID-19 patients could not show impairment of left ventricular (LV) function by conventional ejection fraction (LVEF) [5]. Recent retrospective studies revealed that either left or right ventricular dysfunction was observed in longitudinal strain measurements, possibly predicting mortality in COVID-19 patients [6, 7]. So far, advanced three-dimensional and strain echocardiography (3DSE) has been lacking as part of the comprehensive clinical evaluation of COVID-19 patients.

In this prospective study, we evaluated left and right heart anatomy in COVID-19 patients with and without myocardial injury by comprehensive 3DSE to reveal different patterns of myocardial dysfunction. Additional follow-up 3DSE was used to clarify whether the observed myocardial dysfunction was transient in COVID-19 patients.

## Methods

### Study population

Patients with confirmed COVID-19 were included in this prospective study conducted at the Ludwig-Maximilians-University (LMU) Hospital in Munich, Germany. The following polymerase chain reaction (PCR) assays were used in the accredited routine diagnostics laboratory of the Department of Virology in order to confirm SARS-CoV-2 infection: the nucleocapsid (N1) reaction of the CDC protocol, the envelope amplification of the Charité protocol, the nucleocapsid amplification of the Seegene Allplex 2019-nCoV Assay, the Roche Cobas SARS-CoV-2 nucleocapsid reaction or the Xpert Xpress SARS-CoV-2 run on the GeneXpert System as previously described [8].

All patients are part of the COVID-19 Registry of the LMU University Hospital Munich (CORKUM, WHO trial ID DRKS00021225). Patients gave written informed consent for participation and analysis of their data. The study was approved by the local ethics committee (No: 20-245) and complies with the Declaration of Helsinki. Data collection contained patient characteristics, laboratory values, echocardiographic findings, and outcome which were recorded in the designated COVID-19 database.

We excluded patients from echocardiographic analysis, in which an elevation of hsTNT was biased by markedly elevated creatinine levels, previous or ongoing need for dialysis, or a high likelihood of pulmonary embolism as the cause of myocardial damage. Furthermore, patients with high suspicion of myocardial ischemia due to acute coronary syndromes, including ST segment alterations, were also excluded from the echocardiographic analysis. If patients had insufficient acoustic windows, e.g. due to prone position, they were also excluded.

### Definitions

COVID-19 was confirmed by positive RT-PCR for SARS-CoV-2 from throat swabs, sputum or endotracheal suction. Presence of myocardial injury was defined by elevated hsTNT levels (Elecsys Troponin T hs by Roche Diagnostics GmbH, Vienna, Austria) above the 99th percentile upper reference limit (0.014 ng/ml). Acute respiratory distress syndrome (ARDS) was diagnosed according to the Berlin definition and Horovitz Index served for differentiation of severity as described before [5]. Pre-existing cardiovascular disease (CVD) comprised coronary artery disease, atrial fibrillation or known heart failure.

Systolic LV dysfunction (LVD_sys_) was indicated if 3D-LVEF was below 50% [9, 10], or if LV global longitudinal strain (LV-GLS) was above -16% [11]. Diastolic LV dysfunction (LVD_dia_) was present if three out of the following six parameters were found: Mitral peak *E* velocity ≤ 50 cm/s, *E*/*A* ratio ≤ 0.8, average *E*/*e*′ > 14, septal *e*′ velocity < 7 cm/s or lateral *e*′ velocity < 10 cm/s, tricuspid regurgitation (TR) velocity > 280 cm/s or left atrial volume (3D-LAV) index > 34 ml/m^2^ [12]. Systolic right ventricular (RV) dysfunction (RVD_sys_) was defined as 3D-RVEF below 45% [13] or impaired RV free wall strain (RV-FWS) above -20% [9].

### Echocardiography

Initial comprehensive transthoracic echocardiography was performed on normal ward or intensive care unit (ICU) within 5–21 days after admission. Follow-up echocardiography took place in an outpatient setting. A X5-1 transducer on a EPIQ CVx cardiac ultrasound system (Philips Healthcare, Andover, MA) was used exclusively for COVID-19 patients during the study period. Data acquisition and analysis followed current recommendations for the assessment of native valve regurgitation and chamber quantification [14, 15]. LV linear dimensions (LV end-diastolic and end-systolic diameters) were measured in parasternal long axis. LV volumes (end-diastolic, end-systolic), 3D-LVEF, LV mass, left atrial end-systolic volume (3D-LAV) were measured with the semi-automatic 3D HeartModel tool (Philips Healthcare, Andover, MA). LV forward stroke volume was quantified with the Doppler velocity time integral method in the LV outflow tract. Transmitral pulse wave Doppler and tissue Doppler imaging were used to quantify diastolic function with *E*/*A* and average *E*/*e*′ ratio and septal as well as lateral wall movement. Myocardial contraction fraction (MCF) represents the ratio of stroke volume and myocardial volume (MV). MV was estimated by dividing LV mass by density (1.05 g/ml) [16]. LV global longitudinal strain (LV-GLS) analysis was performed with AutoStrain function (TOMTEC Imaging Systems, Unterschleissheim, Germany). Assessment of right ventricular (RV) dimension and function was performed with the 4D RV-FUNCTION tool (TOMTEC Imaging Systems, Unterschleissheim, Germany) [17], including 3D RV volumes (end-diastolic, end-systolic), 3D-RVEF, global RV stroke volume, tricuspid annular plane systolic excursion (TAPSE), RV mid-ventricular diameter and length, fractional area change (FAC) and RV-FWS. Right atrial (RA) end-systolic volume was calculated by single plane area-length method. We measured systolic tricuspid regurgitation (TR) peak gradient using the simplified Bernoulli equation. By adding the estimated RA pressure to the systolic TR peak gradient, we calculated the systolic pulmonary artery pressure (Echo-sPAP).

### Statistical analysis

Data are expressed as median with interquartile range (IQR) or number (*n*) with percentage of total. Continuous variables were checked for normal distribution with Kolmogorov–Smirnov-test. Student’s *t* test was applied for normally distributed, continuous variables; otherwise Mann–Whitney *U* test was used. Wilcoxon test was employed for pairwise comparison of variables between initial and follow-up measurements. Chi-square (χ^2^) test was employed for comparison of categorical variables. Pearsons’ *r* was used to assess bivariate correlation between different variables. Interobserver variability for exemplary measurements of LV end-diastolic diameter and TAPSE were obtained by analysis of 20 random patients by two independent echocardiographers. The results were analysed using intraclass correlation coefficient (ICC). Statistical significance was considered as of *p* value <0.05. All study variables were analysed using SPSS statistical software (IBM, USA, version 26) or graph pad prism software (GraphPad Software, USA, version 8).

## Results

### Patient characteristics of hospitalised COVID-19 patients with and without myocardial injury

Between February 28th and May 7th 2020, 116 patients were hospitalised for symptomatic COVID-19 disease. Patients were screened for suspected myocardial injury according to elevated hsTNT level. 76 patients were excluded due to elevated creatinine levels as sign of severe kidney failure or intermittent need for dialysis, markedly elevated D-Dimer with high suspicion for thromboembolism, electrocardiographic ST-segment changes as sign for (non-) ST-elevation myocardial infarction or missing patient consent. Echocardiography was performed in 40 patients, of whom eight patients were additionally excluded due to inadequate acoustic windows. Patients without elevated hsTNT were included to serve as control group. A total of 32 patients with comprehensive 3DSE were included in this analysis, 18 with suspected myocardial injury (hsTNT+) and 14 without myocardial injury (hsTNT-).

Patients characteristics and laboratory findings are depicted in Table 1. The majority of patients (88%) were male without significant difference between groups (94% in hsTNT+ vs. 79% in hsTNT-, *p* = 0.178). Patients with myocardial injury were older (68 years vs. 53 years, *p* = 0.001) and suffered from more comorbidities in comparison with hsTNT-patients. The study groups did not differ in terms of intake of medication. Newly started medication during the inpatient stay was comparable in both groups, although some patients were started on ACE-inhibitors, ARB/ARNI or betablockers as a response to echocardiographic findings on myocardial injury. Overall, 7 hsTNT+ patients but no hsTNT- patient received hydroxychloroquine at the discretion of the treating physician (*p* = 0.009).

**Table 1:** Clinical characteristics

Number (%) or median (IQR)	All (*n* = 32)	With myocardial injury (*n* = 18)	Without myocardial injury (*n* = 14)	*p*-value
Age, years	62 (53–72)	68 (61–77)	53 (49–59)	**0.001**
Male sex	28 (88%)	17 (94%)	11 (79%)	0.178
Duration of hospitalisation, days	20 (10–33)	21 (17–34)	12 (8–29)	0.084
Time “Onset of symptoms to evaluation”, days	15 (11–27)	20 (7–29)	15 (12–21)	0.543
Time “Admission to evaluation”, days	11 (5–16)	12 (6–25)	10 (5–14)	0.253
Comorbidities				
Pulmonary artery embolism in CT- angiography, number of CT-scans	1 (*n* = 9)	1 (*n* = 6)	0 (*n* = 3)	0.453
Hypertension	17 (53.1%)	13 (72.2%)	4 (28.6%)	**0.016**
Cardiovascular disease	8 (25.0%)	7 (38.9%)	1 (7.1%)	**0.043**
Diabetes mellitus	7 (21.9%)	5 (27.8%)	2 (14.3%)	0.367
Chronic lung disease	5 (15.6%)	4 (22.2%)	1 (7.1%)	0.251
Medication				
Any medication of the following, *n*	18 (56.3%)	11 (61.1%)	7 (50%)	0.539
ACE-inhibitors	9 (28.1%)	6 (33.3%)	3 (21.4%)	0.457
ARB/ARNI	4 (12.5%)	2 (11.1%)	2 (14.3%)	0.788
Betablocker	11 (34.4%)	7 (38.9%)	4 (28.6%)	0.542
Hydroxychloroquine	7 (21.9%)	7 (38.9%)	0	0.009
Start of medication during admission, *n*	7 (21.9%)	5 (27.8%)	2 (14.3%)	0.360
Laboratory findings				
Initial hsTNT, ng/ml	< 0.013 (< 0.013–0.022)	0.021 (0.014–0.031)	< 0.013	** < 0.001**
hsTNT on evaluation, ng/ml	0.075 (< 0.013–0.031)	0.030 (0.017–0.074)	< 0.013	** < 0.001**
Maximum hsTNT, ng/ml	0.017 (< 0.013–0.045)	0.040 (0.023–0.150)	< 0.013	** < 0.001**
Time to maximum hsTNT after admission, days		17 (8.8–25.8)		
Serum creatinine on evaluation, mg/dl	0.8 (0.7–1.0)	0.9 (0.8–1.2)	0.8 (0.6–0.9)	**0.026**
D-dimer on evaluation, µg/ml	2.25 (0.78–4.55) (*n* = 18)	2.25 (0.70–4.23) (*n* = 12)	3.10 (0.78–6.3) (*n* = 6)	**0.606**
Maximum NT-proBNP, ng/ml	410 (138–919)	744 (260–1,257)	117 (23–416)	0.001
ICU treatment				
Admission on ICU, *n*	15 (46.9%)	11 (61.1%)	4 (28.6%)	**0.031**
Duration of ICU treatment, days	15 (5–21)	17 (5–21)	13 (7–23)	0.793
Time admission ICU to echo evaluation, days	11 (6–24)	16 (6–26)	9 (3–14)	0.327
Intubation with mechanical ventilation, *n*	14 (43.8%)	10 (55.6%)	4 (28.6%)	0.133
Minimal Horovitz Index (P/F-ratio), mmHg	125.5 (101.0–139.3)	121.0 (80.8–139.3)	125.5 (110.0–228.8)	0.620
ARDS severity, *n*				
Mild	3 (21.4%)	2 (20%)	1 (25%)	0.469
Moderate	10 (71.4%)	7 (70%)	3 (75%)	0.852
Severe	2 (14.3%)	2 (20%)	0	0.179
Intubated at the time of echo evaluation, *n*	3	2	1	0.770

ACE-inhibitors, angiotensin-converting enzyme inhibitors; ARB, angiotensin II receptor blocker; ARNI, angiotensin receptor neprilysin inhibitor; CT, computed tomography; echo, echocardiography; hsTNT, high sensitive troponin T; NT-proBNP, N-terminal prohormone brain natriuretic peptide; ICU, intensive care unit; P/F-ratio, paO_2_/FiO_2_-ratio, ratio of arterial oxygen partial pressure to fractional inspired oxygen; ARDS, acute respiratory distress syndrome

Peak NT-proBNP levels significantly differed between both groups (744 vs. 117 ng/ml, *p* = 0.001) and serum creatinine levels were slightly higher in hsTNT+ patients (0.9 mg/dl (hsTNT+) vs. 0.8 mg/dl (hsTNT-), *p* = 0.026) but within normal reference intervals.[18] Levels of c-reactive protein and interleukin 6 of both groups were similar.

### Clinical outcome of hospitalised COVID-19 patients with and without myocardial injury

Median duration of hospitalisation was 20 days, ranging from 5 days (minimum) to 73 (maximum) days with tendency of hsTNT+ patients to stay longer in hospital (Table 1). Admission to intensive care unit (61% vs. 29% patients, *p* = 0.031) did statistically differ, whereas need for intubation due to respiratory failure (10 vs. 4 patients, *p* = 0.133) did not statistically differ between groups. Median duration of ICU treatment was 15 (5–21) days. None of the 32 patients died during hospitalisation or time to follow-up.

### Echocardiographic analysis of left and right heart dimensions and function

Although LV dimensions did not differ between groups (Table 2), the RV was enlarged in hsTNT+ patients with RV end-systolic volume of 68 vs. 52 ml in hsTNT-patients (*p* = 0.019). Systolic LV function evaluated by 3D-LVEF was normal and comparable between groups with 52% (hsTNT+) vs. 59% (hsTNT-, *p* = 0.074). We observed a significant impairment of MCF (0.47 vs. 0.55, *p* = 0.016) and LV-GLS (-13.9% vs. -17.7%, *p* = 0.005) in hsTNT+ patients as indicator for systolic left ventricular dysfunction (LVD_sys_). In addition, LVD_dia_ was more frequent in hsTNT+ compared to hsTNT- patients and concomitantly observed in about one half of hsTNT+ patients with systolic ventricular dysfunction.

**Table 2:** Echocardiographic parameters

Number (%) or median (IQR)	All (*n* = 32)	With myocardial injury (*n* = 18)	Without myocardial injury (*n* = 14)	*p*-value
Left ventricular dimensions				
LVEDD, mm	48 (45–51)	48 (46–52)	47 (43–49)	0.208
LVESD, mm	38 (33–42)	40 (34–44)	35 (32–40)	0.165
LV mass, g	143 (125–181)	156 (135–179)	135 (118–197)	0.287
MV, ml	137.6 (121.9–172.6)	148.6 (133.8–171.9)	128.6 (111.9–187.1)	0.196
3D-LVEDV, ml	131 (113–156)	131 (113–147)	131 (117–159)	0.805
3D-LVESV, ml	56.0 (48.3–72.0)	56 (50–76)	56 (41–64)	0.518
3D-LAV, ml	66 (48–93)	77 (48–104)	57 (49–69)	0.119
Systolic left ventricular function				
MCF, ratio	0.50 (0.43–0.57)	0.47 (0.39–0.55)	0.55 (0.49–0.64)	0.016
3D-LVEF, %	55 (50–62)	52 (46–61)	59 (53–64)	0.074
Forward LV SV, ml	72 (58–90)	67 (57–85)	78 (62–93)	0.362
LV-GLS, %	−15.1 (−11.7 to −18.8)	−13.9 (−9.3 to −16.5)	−17.7 (−14.8 to −19.8)	0.005
Diastolic left ventricular function				
* E*/*A*, ratio	0.9 (0.7–1.1)	0.7 (0.7–0.9)	1.1 (0.9–1.2)	0.004
Mitral *E* velocity, cm/s	74.7 (57.1–85.6)	60.0 (50.1–75.9)	80.9 (74.1–87.2)	0.002
Average *E*/*e*′, ratio	7.7 (6.3–9.7)	7.6 (5.9–8.9)	8.2 (6.5–10.5)	0.471
Septal *e*′ velocity, cm/s	7.9 (6.5–9.8)	6.6 (5.4–8.6)	9.6 (7.9–11.1)	0.005
Lateral *e*′ velocity, cm/s	10.6 (8.7–12.8)	9.3 (8.1–10.8)	12.7 (10.1–14.9)	0.017
TR velocity, cm/s	195.0 (139.5–217.0)	199.4 (123.5–214.5)	162.0 (139.5–219.5)	0.790
3D-LAV index, ml/m^2^	36.0 (24.8–47.4)	44.4 (31.6–52.1)	29.6 (23.5–37.6)	0.134
Right ventricular dimensions				
3D-RVEDV, ml	108.0 (89.0–126.5)	112.5 (95.4–127.0)	99.2 (82.5–123.0)	0.305
3D-RVESV, ml	58.2 (46.3–73.5)	67.5 (57.5–77.7)	51.5 (43.2–57.5)	0.019
RV mid, mm	34.5 (31.7–39.0)	34.8 (31.9–39.2)	34.3 (30.5–39.1)	0.704
RV length, mm	75.6 (72.1–80.5)	76.0 (72.4–81.0)	75.6 (71.4–80.9)	0.704
RA volume, ml	44.6 (34.5–62.4)	46.9 (40.7–59.7)	42.9 (33.0–63.7)	0.427
Right ventricular function				
TAPSE, mm	23.5 (19.8–27.0)	26.0 (20.5–28)	22.0 (18.8–25.5)	0.074
FAC, %	40 (35–45)	37 (29–43)	43 (40–48)	0.014
3D-RVEF, %	44 (39–49)	40 (34–44)	49 (46–53)	0.001
Global RV SV, ml	45.4 (39.3–54.0)	43.1 (31.3–53.7)	49.4 (42.7–55.1)	0.154
RV-FWS, %	-24.0 (-17.2- -29.0)	-18.5 (-13.6- -24.6)	-28.3 (-24.2- -32.2)	0.003
TR peak, mmHg	16.5 (9.3–21.5)	17.5 (9.5–26.0)	13.5 (8.5–21.0)	0.487
Echo-sPAP, mmHg	1.19 (0.76–1.85)	1.20 (0.78–1.61)	1.05 (0.76–2.42)	0.935
RV *S′* max, mm/s	16.0 (12.5–18.9)	15.2 (11.9–19.8)	16.0 (12.6–18.2)	0.739
VCI width, mm	15 (13–19)	16 (13–19)	15 (14–19)	0.873

3D, three-dimensional; LVEDD, left ventricular diastolic diameter; LVESD, left ventricular systolic diameter; LV, left ventricle; MV, myocardial volume; LVEDV/RVEDV, left/right ventricular end-diastolic volume; LVESV/RVESV, left /right ventricular end-systolic volume; LA, left atrial volume; MCF, myocardial contraction fraction; LVEF/RVEF, left /right ventricular ejection fraction; SV, stroke volume; GLS, global longitudinal strain; E/A, ratio of mitral E-wave to A-wave; RV, right ventricle; RA, right atrial; TAPSE, tricuspid annular plane systolic excursion; FAC, fractional area change; RV-FWS, right ventricular free wall strain; TR, tricuspid regurgitation; sPAP, systolic pulmonary artery pressure; *S′* max, maximum systolic excursion velocity; VCI, vena cava inferior

Furthermore, systolic dysfunction of the right ventricle was observed more often in patients with myocardial injury than in patients without myocardial injury. In detail, FAC (37% vs. 43%, *p* = 0.014), 3D-RVEF (40% vs. 49%, *p* = 0.001) and RV-FWS (-18.5% vs. -28.3%, *p* = 0.003) were significantly reduced in hsTNT+ patients in contrast to normal values in hsTNT- patients. Troponin levels of patients with myocardial injury did not correlate with 3D-LVEF, LV-GLS, TAPSE, FAC, 3D-RVEF or RV-FWS.

**Fig. 1:**
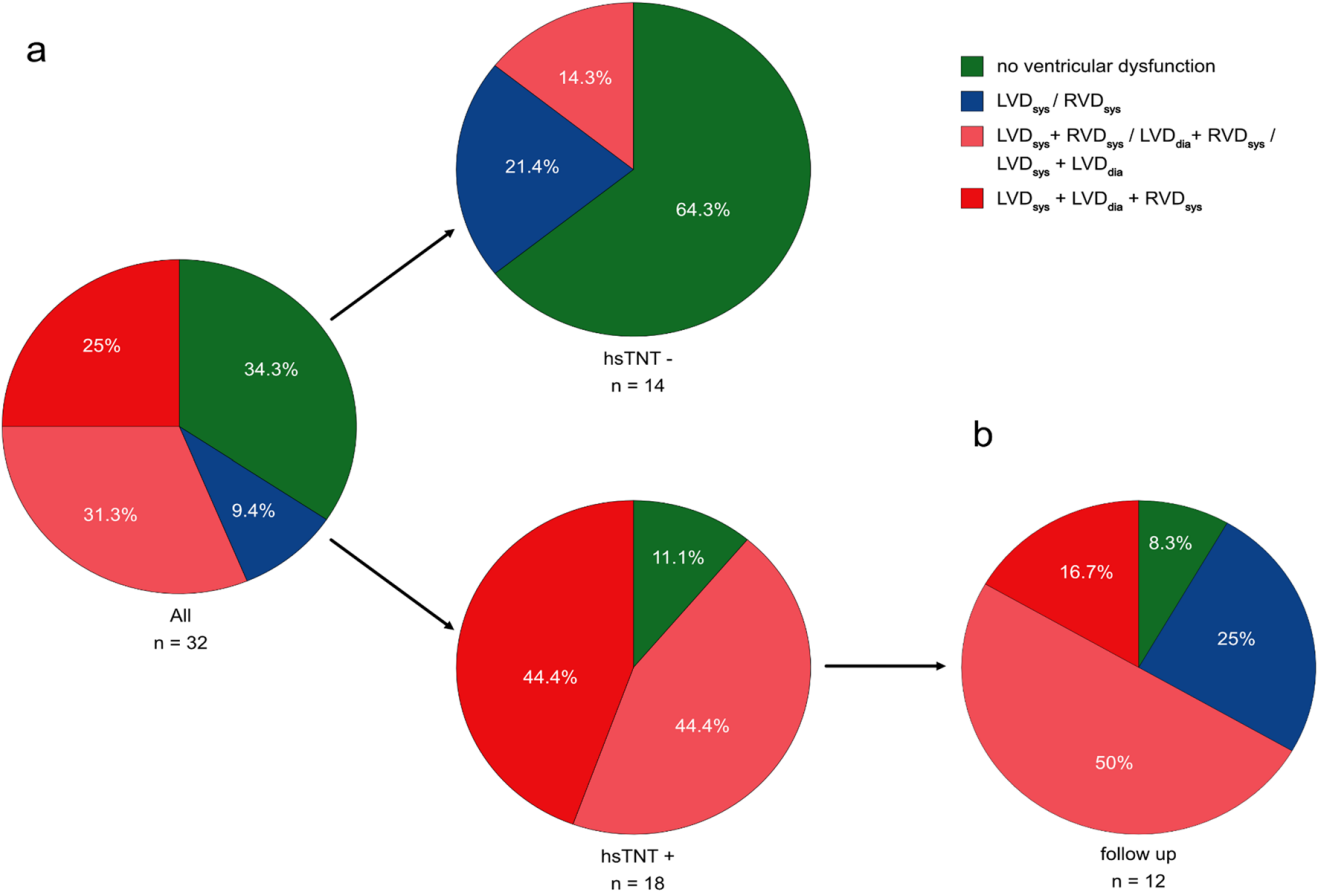
Patterns of ventricular dysfunction in hospitalised patients with COVID-19. (**a**) Distribution of echocardiographic findings in all 32 patients hospitalised with COVID-19 and separated by evidence for myocardial injury according to elevated high-sensitive troponin T (hsTNT). hsTNT+ indicates myocardial injury, hsTNT- denotes no myocardial injury. (**b**) Distribution of echocardiographic findings on follow-up of 12 patients with previous history of COVID-19-associated myocardial injury. LVD_sys_, systolic left ventricular dysfunction; LVD_dia_, diastolic left ventricular dysfunction; RVD_sys_, systolic right ventricular dysfunction; *n*, number of patients

Taken together, combined ventricular heart failure with either systolic biventricular dysfunction (LVD_sys_ + RVD_sys_, LVD_dia_ + RVD_sys_) or systolic and diastolic left ventricular dysfunction (LVD_sys_ + LVD_dia_) or triple dysfunction (LVD_sys_ + RVD_sys_ + LVD_dia_) were present in about 56.3% of all patients and occurred more frequently in hsTNT + than in control (88.8% vs. 14.3%, *p* < 0.001; Fig. 1a).

### Echocardiographic follow-up

**Fig. 2:**
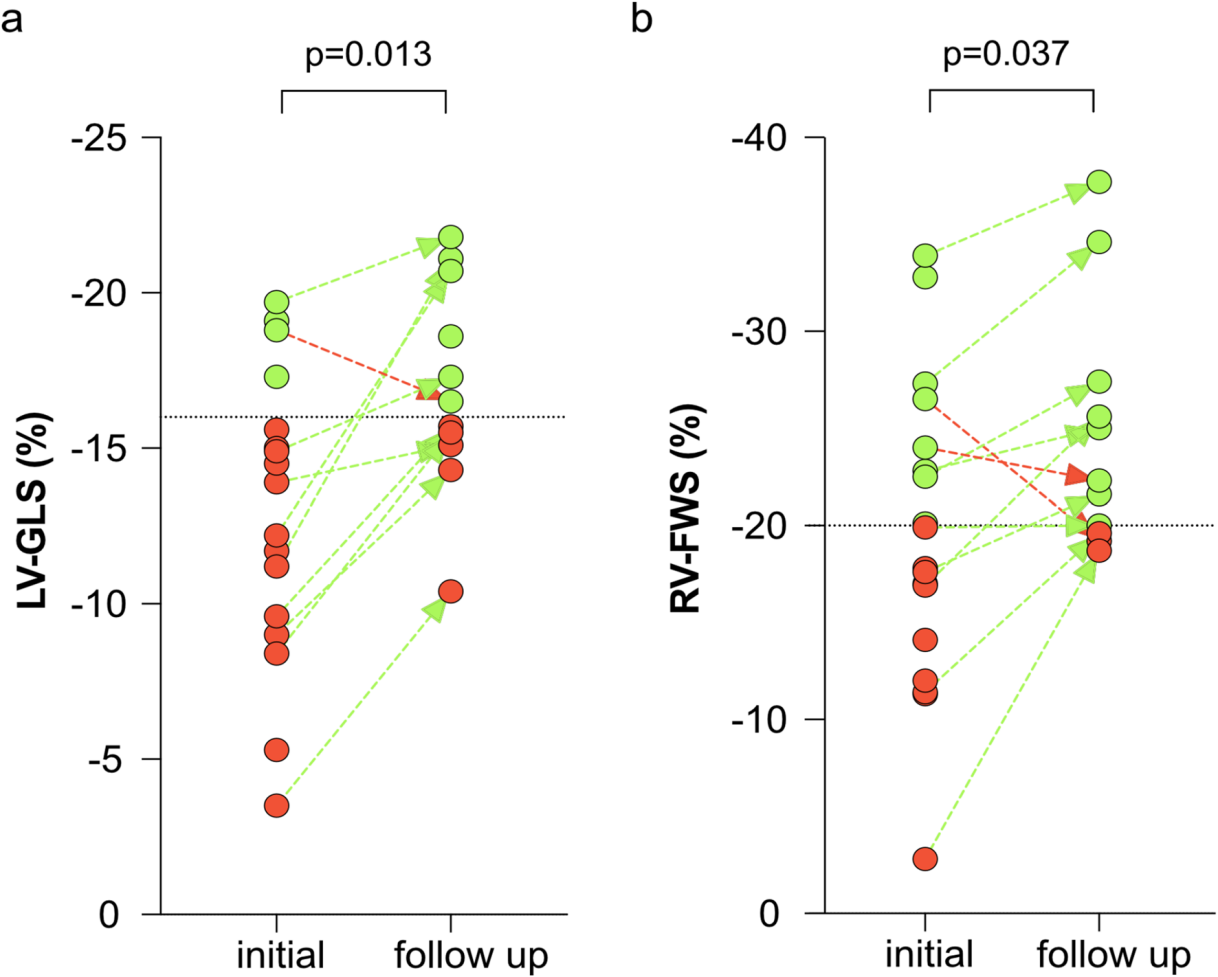
Trend of left ventricular global longitudinal strain and right ventricular free wall strain in patients with myocardial injury. Left ventricular global longitudinal strain (LV-GLS, **a**) and right ventricular free wall strain (RV-FWS, **b**) measurements in patients with COVID-19-associated myocardial injury at initial assessment and on follow-up after a median of 52 days. *Red dots* represent values below the reference value of LV-GLS of -16% (*dotted black line*, **a**) or RV-FWS of -20% (*dotted black line*, **b**), respectively. *Green dots* represent values within normal range. *Dashed arrows* represent improvement (*green*) or deterioration (*red*) of LV-GLS or RV-FWS. LV-GLS, left ventricular global longitudinal strain; RV-FWS, right ventricular free wall strain

Twelve hsTNT+ patients underwent follow-up echocardiography within 52 (47–68) days after initial evaluation. Overall, six (33.3%) hsTNT + patients were lost to follow-up. The percentage of hsTNT + patients suffering from triple ventricular dysfunction (LVD_sys_ + RVD_sys_ + LVD_dia_) decreased from initially 44.4% to 8.3% at follow-up (Fig. 1b), whereas isolated ventricular dysfunction (LVD_sys_ or LVD_dia_ or RVD_sys_) occurred in three (25%) hsTNT+ patients at follow-up. In detail, 3DSE showed that LV-GLS (-13.9% vs. -16.5%, *p* = 0.013) and RV-FWS (-18.5% vs. -22.3%, *p* = 0.037) significantly improved in hsTNT+ patients (Fig. 2). Interestingly, RV diameters as well as global right ventricular stroke volume did increase in hsTNT+ patients upon follow-up in comparison with their initial measurements, but these did not significantly differ (Table S1).

## Discussion

This is the first comprehensive echocardiographic study applying advanced 3D and strain imaging methods in hospitalised COVID-19 patients with and without myocardial injury. We demonstrated that COVID-19 patients frequently present with biventricular dysfunction, which partially resolves within two months after hospital discharge. In our study, patient characteristics, in particular age, hypertension and CVD, were comparable to larger hospitalised COVID-19 patient cohorts [1, 2, 19]. Our study shows that patients with myocardial injury were older, had more comorbidities and CVD, with higher rates of ICU admission and need for mechanical ventilation compared to patients without myocardial injury.

Importantly, conventional assessment of LV function by LVEF did not reveal differences between groups of myocardial injury. In line with the findings of a recent study of Janus et al. [7], we observed that rather LV-GLS than LVEF is the modality of choice to detect systolic left ventricular dysfunction (LVD_sys_) in patients with COVID-19-associated myocardial injury. LVD_sys_ was mainly detected by impaired LV-GLS (12 out of 19 patients with LVD_sys_).

Furthermore, advanced 3DSE revealed that the majority of hospitalised COVID-19 patients had biventricular dysfunction. This pattern was highly prevalent in hsTNT+ patients (>80% of patients). Comparable to LVD_sys_, RVD_sys_ was mainly diagnosed by impaired 3D-RVEF or RV-FWS. Of note, pathological patterns were also found in patients without cardiac preconditions. Severity of ventricular dysfunction or the aforementioned RV enlargement was not associated with the length of hospital or ICU stay or with duration of mechanical ventilation. Interestingly, RV diameters as well as global right ventricular stroke volume did increase in hsTNT+ patients upon follow-up in comparison with their initial measurements, but these did not significantly differ (Table S1). Concerning interobserver variability, ICCs were within acceptable range (LV end-diastolic diameter: 0.862, *p* < 0.001; TAPSE: 0.882, *p* < 0.001).

Importantly, troponin levels of patients with myocardial injury did not correlate with 3D-LVEF, LV-GLS, 3D-RVEF or RV-FWS. These findings support a diagnostic pathway of first identifying myocardial damage by biomarkers with subsequent characterisation by comprehensive 3DSE to reveal the extent and pattern of myocardial impairment that might be underestimated by point-of-care ultrasound or single laptop-based equipment as previously recommended [20]. Especially strain imaging was useful to evaluate biventricular myocardial contractility, revealing subclinical LV dysfunction in our cohort.

Recently, two reports have shown that COVID-19 patients can suffer from RVD_sys_ and RV dilatation [6]. However, in the study of Arguilan et al., 30% of patients were intubated and mechanically ventilated at the time of examination and evidence of pulmonary artery embolism was present in about 50% [21], possibly contributing to RVD_sys_ by increased RV afterload. In our study cohort with myocardial injury, increased afterload was scarce and only one patient had evidence of pulmonary embolism in computed tomography scan.

At follow-up, we detected an improvement of biventricular systolic function in patients with previous COVID-19-associated myocardial injury. In comparison with baseline measurements, LV-GLS and RV-FWS significantly improved. Although we cannot provide echocardiographic data before onset of COVID-19 in these patients, our findings implicate that biventricular dysfunction in patients with COVID-19-associated myocardial injury could be transient and partially or even completely resolve over time.

In clinical survey at follow-up, some patients reported on ongoing respiratory insufficiency, so evaluation of New York Heart Association (NYHA) functional classification was fairly limited in validity. However, NT-proBNP levels significantly improved (744 pg/nl (initial) vs. 130 pg/nl (follow-up), *p* = 0.026), encouraging our implications on transient myocardial dysfunction under COVID-19.

Finally, we observed that with proper precautions including adequate personal protection equipment, comprehensive echocardiographic examinations can be safely performed during the COVID-19 pandemic.

### Limitations

Our study cohort is relatively small but contains the largest comprehensive echocardiographic 3D and strain assessment of biventricular function in COVID-19 patients. Since no COVID-19 patient who underwent comprehensive echocardiography died, no predictors for mortality can be identified. Further, we cannot deduce from our data whether pathological findings in relatively old study cohort are associated with bad outcome in the sense of long-term heart failure. Since no previous comprehensive 3D and strain echocardiographic studies on patients with known cardiovascular disease were performed before the COVID-19 pandemic, we cannot exclude that our findings have existed before assessment.

The aetiology of myocardial injury in COVID-19 is not fully understood and beyond the scope of this study.

## Conclusion

COVID-19 is associated with different patterns of systolic and diastolic biventricular dysfunction in hospitalised patients. The current prospective study shows that advanced 3D and strain echocardiography has superior diagnostic value over conventional 2D echocardiography, revealing LVD_sys_ by LV-GLS and RVD_sys_ by either 3D-RVEF or RV-FWS. The prognostic relevance of concomitant LVD_sys_, RVD_sys_ and LVD_dia_ will need to be further evaluated by endpoint analysis in the future in larger patient numbers. Taken together, comprehensive echocardiographic examinations can serve as a sensitive diagnostic tool to reveal pathologic patterns of myocardial function and their resolution after hospital discharge.

## Supplementary Information

Below is the link to the electronic supplementary material.

Supplementary file1 (PDF 99 KB)

## Data Availability

The data that support the findings of this study are available from the corresponding authors upon request.

## Code availability

Semi-automatic 3D HeartModel tool (Philips Healthcare, Andover, MA); AutoStrain function (TOMTEC Imaging Systems, Unterschleissheim, Germany); 4D RV-FUNCTION tool (TOMTEC Imaging Systems, Unterschleissheim, Germany).
