# Supplementary material for: Left and right ventricular dysfunction in patients with COVID-19-associated myocardial injury
Source: Infection. 2021 Jan 30;49(3):491–500. doi: 10.1007/s15010-020-01572-8 (PMC7846912; doi:10.1007/s15010-020-01572-8)
Supplement: Supplementary file 1 — Supplementary file1 (PDF 99 KB) [file 15010_2020_1572_MOESM1_ESM.pdf]

SUPPLEMENTAL DATA TO

Left and right ventricular dysfunction in patients with COVID-19 associated myocardial injury

Infection

Stephanie Bieber, MD[1,2,#], Angelina Kraechan, MD[1,2], Johannes C. Hellmuth, MD[2,3], Maximilian Muenchhoff, MD[2,4,5], Clemens Scherer, MD[1,2,7], Ines Schroeder, MD[6], Michael Irlbeck, MD[6], Stefan Kaeaeb, MD[2,7], Steffen Massberg, MD[1,7], Joerg Hausleiter, MD[1,7], Ulrich Grabmaier, MD[1,2,7], Mathias Orban, MD[1,7,§] & Ludwig T. Weckbach, MD[1,2,7,8,§,#]

# Correspondence to:
Stephanie Bieber
Medizinische Klinik und Poliklinik I, Klinikum der Universität München
Ludwig-Maximilians-Universität München
Marchioninistraße 15, 81377 Munich, Germany
Phone: +49-89-4400-76085
Email: Stephanie.Bieber@med.uni-muenchen.de
ORCID: 0000-0001-5954-5695

Supplemental Table 1: Echocardiographic findings in COVID-19 patients with myocardial injury initially and on follow up.

Number (%) or median (IQR)	**Initial echocardiography (n = 18)**	**Follow up echocardiography (n = 12)**	**p-value**
**Systolic left ventricular function**			
MCF, ratio	0.47 (0.39-0.55)	0.47 (0.36-0.53)	0.286
3D-LVEF, %	52 (46-61)	55 (52-58)	0.759
LV-GLS, %	-13.9 (-9.3- -16.5)	-16.5 (-15.1- -20.7)	**0.013**
**Diastolic left ventricular function**			
E/A, ratio	0.7 (0.7-0.9)	0.9 (0.7-1.1)	0.140
Mitral E velocity, cm/s	60.0 (50.1-75.9)	64.1 (57.5-71.5)	0.388
Average E/e', ratio	7.6 (5.9-8.9)	8.9 (7.2-10.7)	**0.033**
Septal e' velocity, cm/s	6.6 (5.4-8.6)	7.0 (6.0-8.0)	0.534
Lateral e' velocity, cm/s	9.3 (8.1-10.8)	8.5 (6.3-10.5)	0.424
TR velocity, cm/s	199.4 (123.5-214.5)	180.5 (132.5-229)	0.767
3D-LAV index, ml/m^2	44.4 (31.6-52.1)	36.2 (26-44.1)	0.155
**Right ventricular dimensions**			
3D-RVEDV, ml	112.5 (95.4-127.0)	132.4 (101.8-211.1)	0.074
3D-RVESV, ml	67.5 (57.5-77.7)	76.6 (61.1-102.6)	0.093
**Systolic right ventricular function**			
Global RV SV, ml	43.6 (31.7-54.3)	60.5 (35.8-83.7)	0.139
FAC, %	37 (29-43)	43 (40-48)	0.131
3D-RVEF, %	40 (34-44)	42 (29-50)	0.959
RV free wall strain, %	-18.5 (-13.6- -24.6)	-22.3 (-19.6- -27.4)	**0.037**

MCF, myocardial contraction fraction; 3D, three-dimensional; LV, left ventricle; RV, right ventricle; LVEF/RVEF, left /right ventricular ejection fraction; GLS, global longitudinal strain; E/A, ratio of mitral E-wave to A-wave; RV, right ventricle; RA, right atrial; TAPSE, tricuspid

annular plane systolic excursion; FAC, fractional area change; RV-FWS, right ventricular free wall strain; TR, tricuspid regurgitation; LA, left atrial volume; RVEDV, right ventricular end-diastolic volume; RVESV, right ventricular end-systolic volume; SV, stroke volume.
